# Effect of Donor-Acceptor Concentration Ratios on Non-Radiative Energy Transfer in Zero-Dimensional Cs_4_PbBr_6_ Perovskite/MEH-PPV Nanocomposite Thin Films

**DOI:** 10.3390/polym12020444

**Published:** 2020-02-13

**Authors:** Bandar Ali Al-Asbahi, Saif M. H. Qaid, Abdullah S. Aldwayyan

**Affiliations:** 1 Department of Physics & Astronomy, College of Science, King Saud University, Riyadh 11451, Saudi Arabia; sqaid@ksu.edu.sa (S.M.H.Q.); dwayyan@ksu.edu.sa (A.S.A.); 2 Department of Physics, Faculty of Science, Sana′a University, Sana′a P.O. Box 12544, Yemen; 3 Department of Physics, Faculty of Science, Ibb University, Ibb P.O. Box 70270, Yemen; 4 King Abdullah Institute for Nanotechnology, King Saud University, Riyadh 11451, Saudi Arabia

**Keywords:** 0-D Cs_4_PbBr_6_ perovskite, MEH-PPV, optical properties, donor/acceptor, energy transfer

## Abstract

Composite materials with different concentration ratios of a hybrid of zero-dimensional (0-D) Cs_4_PbBr_6_ perovskite, which acts as a donor (D), and poly[2-methoxy-5-(2-ethylhexyloxy)-1,4-phenylenevinylene] (MEH-PPV), which acts as an acceptor (A), were successfully prepared via a solution blending method prior to being deposited onto glass substrates by a spin-coating technique. The influence of acceptor content on the structural, optical, and energy transfer properties of the donor was investigated. The perovskite nanocrystals formed thin films without any chemical interactions within a matrix of MEH-PPV in the blend. The possibility of dipole–dipole (non-radiative) energy transfer from the 0-D Cs_4_PbBr_6_ to the MEH-PPV was proven. The energy transfer parameters such as *R_o_* (critical distance of the energy transfer), k_app_ (apparent quenching constant), $\emptyset_{DA}$ (quantum yield of D in the presence of A), $\tau_{DA}$ (lifetime of D in the presence of A), *P_DA_* (probability of energy transfer), *η* (efficiency of energy transfer), *R_DA_* (energy transfer radius), *k_ET_* (energy transfer rate constant), *T_DR_* (total decay rate), *A_o_* (critical concentration of A), and *A_π_* (conjugation length) were calculated based on the absorption and emission measurements.

## 1. Introduction

The good electronic properties of halide materials with a perovskite crystal structure [1] have attracted the attention of researchers who have employed these materials as functional material layers in numerous devices, such as quantum-dot light-emitting diodes (QDLEDs) [2], solar cells [3], photodetectors [4], and resistive random access memory (ReRAM) [5]. Moreover, these materials have been widely used in the fabrication of optoelectronic and photonic thin-film devices [6,7] because of their unique properties, such as the ability to tune their band gap energy by manipulating the halide composition, and their high solubility in polar organic solvents, which enables facile deposition processing. However, a major challenge that needs attention is the chemical and electrical stability of hybrid perovskite materials under ambient atmosphere in typical optoelectronic devices [8,9]. Several solutions have been proposed to overcome this problem, including enhancing the morphology of thin films by a coating engineer [10], encapsulation with moisture-resistant layers [11,12], and mixing and changing the acceptor (A) site cation, with the most common materials being of the form MAPbX_3_ (methylammonium lead halide) [13].

The incorporation of conjugated polymers as a matrix layer in optoelectronic devices based on perovskites can play a significant role in enhancing the morphology of the films resulting in superior device performance [14,15]. Moreover, through-space or dipole–dipole energy transfer plays a significant role in the donor/acceptor hybrids that have been used to produce optoelectronic devices with high performance efficiency [16,17,18,19].

In the current work, zero-dimensional (0-D) Cs_4_PbBr_6_ perovskite—acting as a donor (D)—and poly [2-methoxy-5-(2-ethyl-hexyloxy)-1,4-phenylene-vinylene] (MEH-PPV)—acting as an acceptor (A)—were blended with various A/D ratios by the solution blending method. Then, these blends were prepared as thin films using a spin-coating technique. The structural, morphology, and optical properties, and energy transfer mechanisms of the hybrids of 0-D Cs_4_PbBr_6_/MEH-PPV were investigated for the first time, to the best of our knowledge.

## 2. Experimental Details

### 2.1. Materials

Poly [2-methoxy-5-(2-ethyl-hexyloxy)-1,4-phenylene-vinylene] (MEH-PPV, *Mw* = 40,000 g/mol) was purchased from Sigma Aldrich (Saint Louis, MO, USA) and used as received without further purification. High-quality green-emitting zero-dimensional Cs_4_PbBr_6_, synthesized by a simple solution-processed chemical method [20] was purchased from Quantum Solutions LLC, King Abdullah University of Science and Technology (KAUST), Kingdom of Saudi Arabia. A toluene solution with a purity of 99.8%, which was produced by Fluka (Buchs, Switzerland), was used to dissolve all the materials.

### 2.2. Methods

The solution blending method was employed to prepare hybrids of zero-dimensional Cs_4_PbBr_6_ and MEH-PPV, which act as the donor and acceptor, respectively. Solutions of various concentration ratios of A/D (where each A and D in mg/ml concentration), namely 0, 0.1, 0.2, 2.0, and 4.0, were prepared. Then, 50 µL samples of each solution were deposited onto glass substrates using a spin-coating technique (2000 rpm for 20 s). All the thin films were annealed at 120 °C in a vacuum oven to remove any remaining solvent.

### 2.3. Characterization

A Panalytical X’PERT Pro powder X-ray diffractometer (Panalytical, Almelo, The Netherlands) in the θ–2θ geometry was employed to characterize the sample structure. A Cu-Kα radiation source operated at 40 mA and 45 kV was used in the X-ray diffraction (XRD) measurements, and the scanning angle (2θ) was varied from 10° to 80° with a step size of 0.02° at 0.5° min^−1^. The morphology of the 0-D Cs_4_PbBr_6_ and the A/D thin films were investigated by scanning electron microscopy (SEM) (JEOL-7600F, JEOL, Akishima, Japan).

The optical properties of the thin films were investigated by measuring UV–Visible absorption spectra and photoluminescence emission spectra. The UV–Visible spectra were recorded using a V-670 absorption spectrometer (JASCO, Cremella, Italia), whereas the emission spectra were examined using an FP-8200 spectrofluorometer (JASCO, Cremella, Italia) with an excitation wavelength of 350 nm. All the measurements were carried out in the ambient atmosphere.

## 3. Results and Discussion

### 3.1. Structural and Morphology of the Hybrid Thin Films

**Figure 1:**
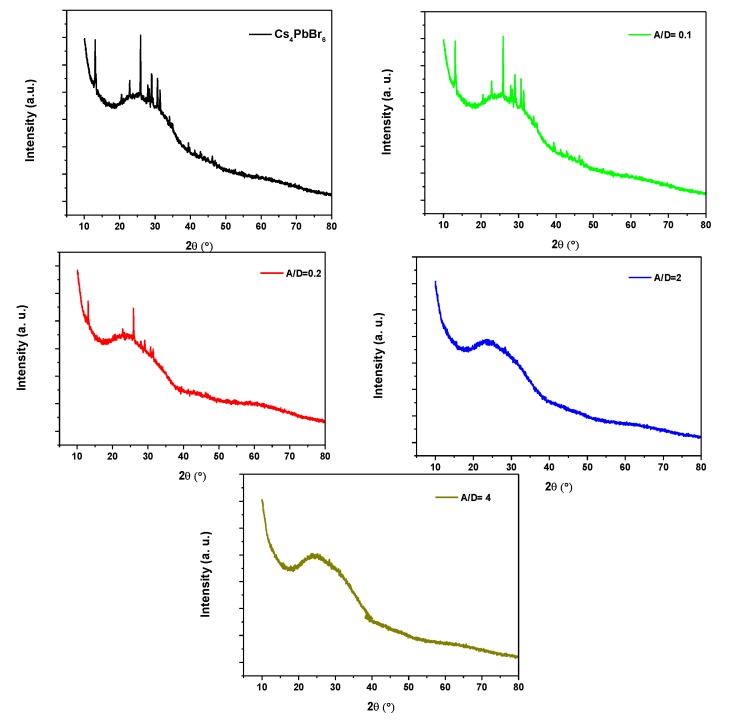
XRD diffractograms of 0-D Cs_4_PbBr_6_ perovskite thin films with various 2-methoxy-5-(2-ethyl-hexyloxy)-1,4-phenylene-vinylene (MEH-PPV) ratios.

Figure 1 shows the XRD patterns of the 0-D Cs_4_PbBr_6_ perovskite thin films with various MEH-PPV ratios (0, 0.1, 0.2, 2.0, and 4.0). The diffraction peaks of the 0-D Cs_4_PbBr_6_ perovskite can be indexed at 2θ = 12.8, 22.7, 25.9, 28.9, 30.5, and 31.4°, which correspond to the (110), (300), (024), (214), (223), and (006) planes. These peaks prove that the 0-D Cs_4_PbBr_6_ perovskite exhibited a rhombohedral structure in the R-3c space group (a = b = 13.73 Å, c = 17.32 Å, α = β = 90°, γ = 120°) at ambient conditions [20,21,22]. These peaks were dramatically reduced in intensity upon increasing the MEH-PPV content and were completely inhibited when the A/D ratio was ≥2.0, where the broadening peak, which corresponds to the amorphous phase of the MEH-PPV, becomes dominant.

**Figure 2:**
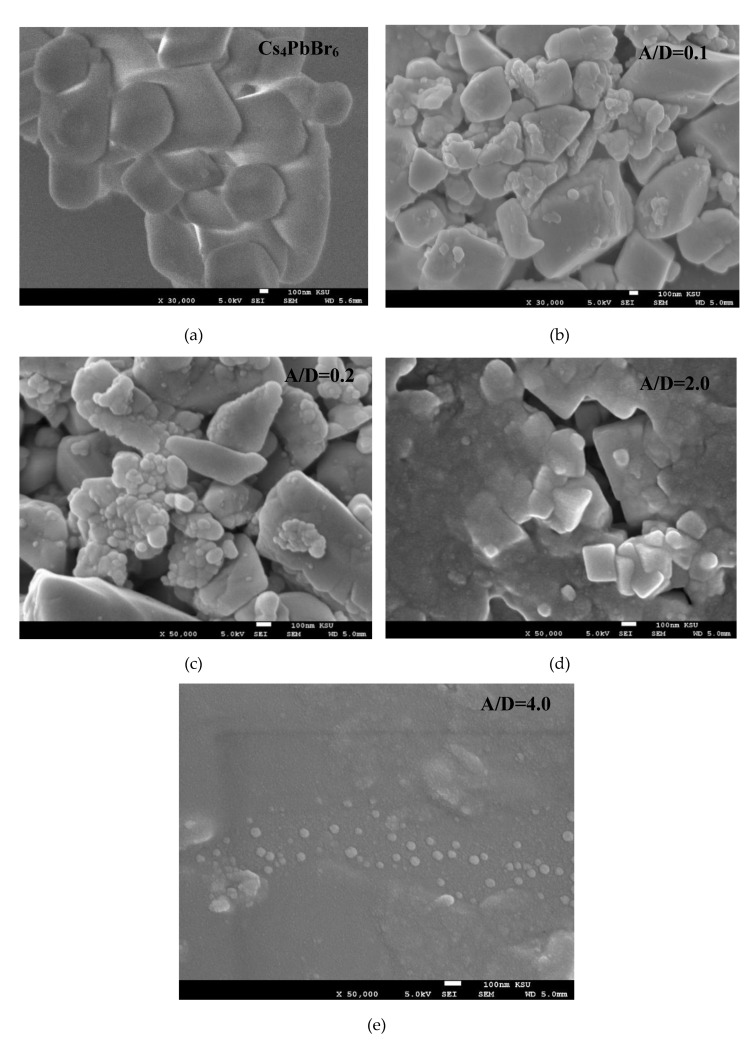
SEM images of the pristine 0-D Cs_4_PbBr_6_ and Cs_4_PbBr_6_/MEH-PPV hybrid thin films with various ratios. (**a**) pristine 0-D Cs_4_PbBr_6_; (**b**) 0.1; (**c**) 0.2; (**d**) 2.0; (**e**) 4.0.

The morphology of the pristine 0-D Cs_4_PbBr_6_ and Cs_4_PbBr_6_/MEH-PPV hybrid thin films prepared with A/D ratios of 0.1, 0.2, 2.0, and 4.0 was investigated using SEM images, as shown in Figure 2. The image in Figure 2a shows that the 0-D Cs_4_PbBr_6_ perovskite consists of rhombohedral nanocrystals approximately 400 nm in dimension. It can be clearly seen that the perovskite crystals are embedded in a uniform layer of the MEH-PPV (Figure 2b–d). While in the thin film with an A/D ratio of 2.0 (Figure 2c) the MEH-PPV layer is incomplete in certain areas; the thin film with an A/D ratio of 4.0 in Figure 2d displays complete MEH-PPV coverage with no signs of pinholes. Consequently, it can be deduced that the perovskite nanocrystals formed within a matrix of MEH-PPV in the hybrid thin films.

### 3.2. Absorption and Fluorescence Spectra of the Thin Films

**Figure 3:**
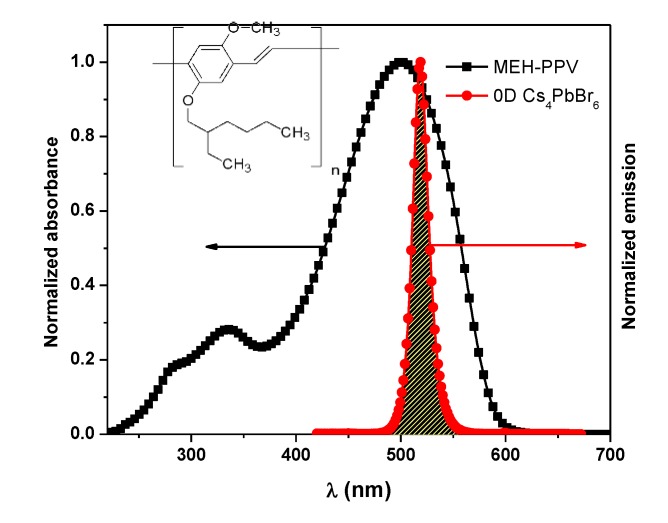
Overlapping between absorption and fluorescence spectra of MEH-PPV and 0-D Cs_4_PbBr_6_. Inset: chemical structure of MEH-PPV.

The absorption and fluorescence spectra of MEH-PPV and 0-D Cs_4_PbBr_6_ are presented in Figure 3. The significant overlap between these spectra provides evidence for the possibility of dipole–dipole (non-radiative) energy transfer from the 0-D Cs_4_PbBr_6_ (donor) to the MEH-PPV (acceptor), which will be investigated in detail in the next section.

**Figure 4:**
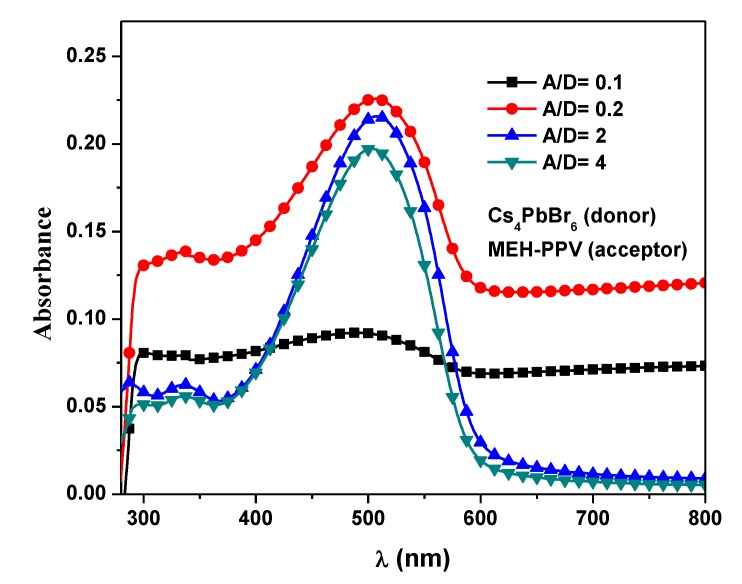
Absorption spectra of the 0-D Cs_4_PbBr_6_/MEH-PPV hybrid thin films.

The absorption spectra of the 0-D Cs_4_PbBr_6_/MEH-PPV hybrid thin films, corresponding to ratios of 0.1, 0.2, 2.0, and 4.0, are illustrated in Figure 4. No new absorbance peak was observed upon increasing the acceptor content, indicating that no aggregates or dimers were formed in the hybrids and no chemical interactions occurred. The absorbance in the wavelength range 600–800 nm, that related to the absorbance of the 0-D Cs_4_PbBr_6_ [23], was significantly reduced upon increment the MEH-PPV content. Addition of MEH-PPV to Cs_4_PbBr_6_ shows systematically decrease in broadening absorption spectra in addition to different trend behavior in the absorbance over the entire wavelength range. This observation can be attributed to decreasing conjugation length and possibility of the occurrence of both static and dynamic quenching, as proved here later.

**Figure 5:**
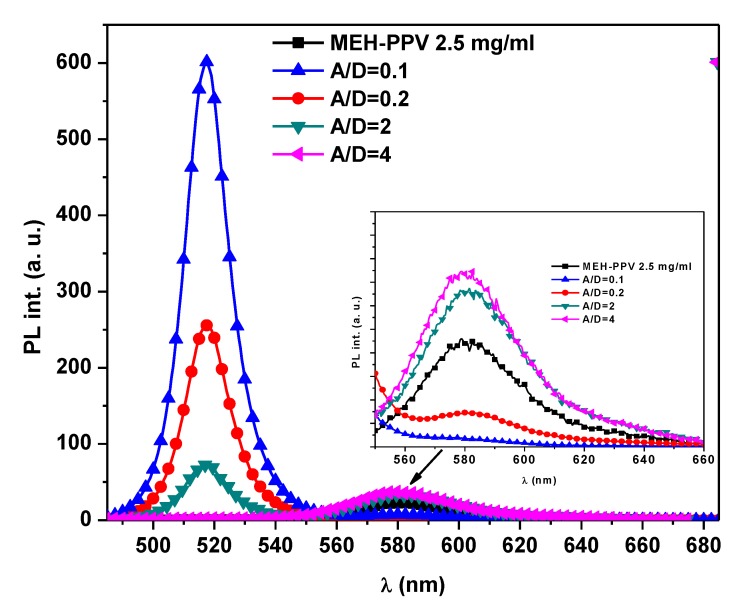
Fluorescence spectra of pristine MEH-PPV, and 0-D Cs_4_PbBr_6_/MEH-PPV hybrid thin films.

The fluorescence spectra of pristine MEH-PPV and the 0-D Cs_4_PbBr_6_/MEH-PPV hybrid thin films are shown in Figure 5. When using an excitation wavelength of 350 nm, the main peaks of MEH-PPV and 0-D Cs_4_PbBr_6_ were detected at 580 and 518 nm, respectively, corresponding to the 0-0 vibrionic transition for each. This excitation wavelength of 350 nm was mainly absorbed by the 0-D Cs_4_PbBr_6_. As the A/D ratio increased, the 0-D Cs_4_PbBr_6_ intensity decreased significantly while the related peak intensity of MEH-PPV increased. This observation confirms the possibility of energy transfer from 0-D Cs_4_PbBr_6_ to MEH-PPV, as acceptor emission made no major contribution under direct excitation at 350 nm.

### 3.3. Energy Transfer Mechanism

In this study, the possibility of non-radiative energy transfer (Förster-type) can be proven based on four main aspects, namely: (i) the strong overlap (Figure 3) between the fluorescence spectrum of 0-D Cs_4_PbBr_6_ perovskite and the absorption spectrum of MEH-PPV; (ii) the strong reduction of the fluorescence intensity of 0-D Cs_4_PbBr_6_ perovskite upon the addition of MEH-PPV (as shown in Figure 5); (iii) the improvement of the fluorescence intensity of MEH-PPV (as shown in Figure 5); and (iv) the critical transfer distance between the molecules of 0-D Cs_4_PbBr_6_ and MEH-PPV, as calculated later. To describe the energy transfer mechanism between 0-D Cs_4_PbBr_6_ and MEH-PPV, various parameters were calculated, such as *R_o_* (critical distance of the energy transfer), k_app_ (apparent quenching constant), $\emptyset_{DA}$ (quantum yield of D in the presence of A), $\tau_{DA}$ (lifetime of D in the presence of A), *P_DA_* (energy transfer probability), *η* (energy transfer efficiency), R_DA_ (energy transfer radius), *k*_ET_ (energy transfer rate constant), *T_DR_* (total decay rate), *A_o_* (critical concentration of acceptor), and *A_π_* (conjugation length).

The following formula can be employed to calculate the critical distance [24]:

(1) $$R_{o}^{6}\;=\;\frac{9000(\ln10)\beta^{2}\phi_{D}}{128\pi^{5}n^{4}N_{o}}\int F_{D}\left(\lambda \right)\varepsilon_{A}\left(\lambda \right)\;\lambda^{4}d\lambda \;\;=\;\frac{9000(\ln10)\beta^{2}\phi_{D}}{128\pi^{5}n^{4}N_{o}}\;J\left(\lambda \right)$$

where *n* is the solvent refractive index, *N*_o_ is Avogadro’s number, *β*^2^ is the orientation factor (0.67 for isotropic media), $\emptyset_{D}$ is the donor quantum yield, *λ* is the wavelength, *ε_A_*(*λ*) is the molar decadic extinction coefficient of the acceptor, and *F_D_*(*λ*) is the normalized spectral distribution of the donor (i.e., **∫***F_D_ (λ*) d*λ* = 1). The *J*(*λ*) and *R_o_* values are tabulated in Table 1. The average *R_o_* was 77.54 Å, which confirmed the domination of non-radiative energy transfer (Förster type) in the blends, where the Förster type is typically effective in the range of 10–100 Å [25,26]. Subsequently, as reported by other researchers, this finding confirms the suitability of Förster theory for calculating the energy transfer parameters [27,28,29].

**Figure 6:**
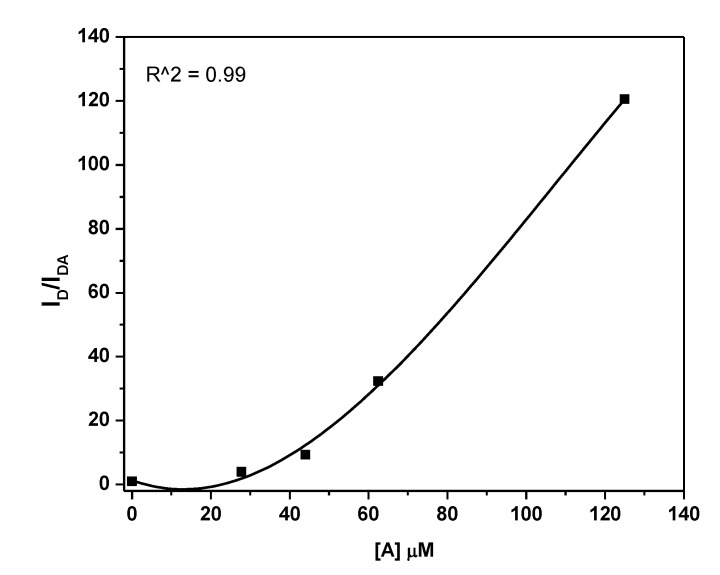
Linear Stern–Volmer plot for 0-D Cs_4_PbBr_6_/MEH-PPV hybrid thin films.

Moreover, the non-radiative energy transfer parameters of the A/D system can be calculated by analyzing its absorption and emission spectra. The following Stern–Volmer equation can be employed to check the type of the emission quenching [30]:

(2) $$\frac{I_{D}}{I_{DA}}\;=\;1+k_{app}\left[A\right],\;\;\;k_{app}\;=\;\left(k_{D}+k_{S}\right)+k_{D}k_{S}\left[A\right]$$

where *I_DA_* and *I_D_* are the emission intensities of D in the presence and absence of A, respectively, $k_{D}\;\&\;k_{S}$ are dynamic and static quenching constants, respectively, and [A] is the acceptor concentration. In many cases, the fluorophore can be quenched both by dynamic and static quenching. A linear Stern–Volmer plot indicates that only one type of quenching occurs, otherwise both types of quenching can occur [30]. Hence, the clear upward curvature shown in Figure 6 indicates that the 0-D Cs_4_PbBr_6_ emission is decreased in the presence of MEH-PPV by both static and dynamic quenching. By fitting the data of Figure 6 using the OriginPro 8 program, a theoretical equation was suggested to find the relationship between $\frac{I_{D}}{I_{DA}}$ and acceptor content as follows:

(3) $$\frac{I_{D}}{I_{DA}}\;=\;1+0.444\left[A\right]+0.0183{\left[A\right]}^{2}$$

By comparing this equation with the Stern–Volmer equation, it can be found that $k_{D}+k_{S}\;=\;0.444\;\;{\mu M}^{-1}$ and $\;k_{D}k_{S}\;=\;0.0183\;\;{\mu M}^{-2}$. The solutions of these equations are $\;k_{D}\;=\;0.40\;\;{\mu M}^{-1}\;\&\;\;k_{s}\;=\;0.05\;\;{\mu M}^{-1}.$ This indicates that at a MEH-PPV concentration of 20 µM, 50% of the ground-state 0-D Cs_4_PbBr_6_ is complexed, and thus is non-fluorescent.

The *ϕ_DA_* and *τ_DA_* values of the hybrid thin films are listed in Table 1. The significant decreases in both values with the addition of MEH-PPV compared with those of the pristine 0-D Cs_4_PbBr_6_ thin film (*ϕ_D_* = 0.45 and *τ_D_* = 5.3 ns) [21] provide theoretical evidence of efficient energy transfer from 0-D Cs_4_PbBr_6_ to MEH-PPV.

**Figure 7:**
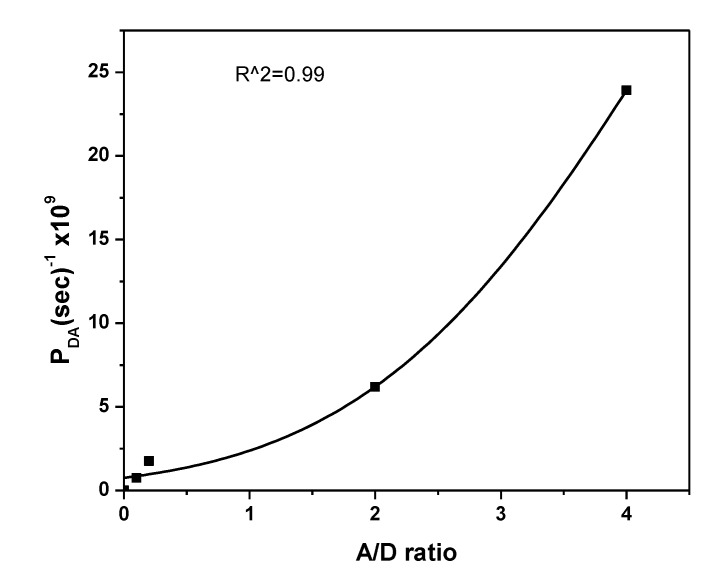
Energy transfer probability for 0-D Cs_4_PbBr_6_/MEH-PPV hybrid thin films.

**Figure 8:**
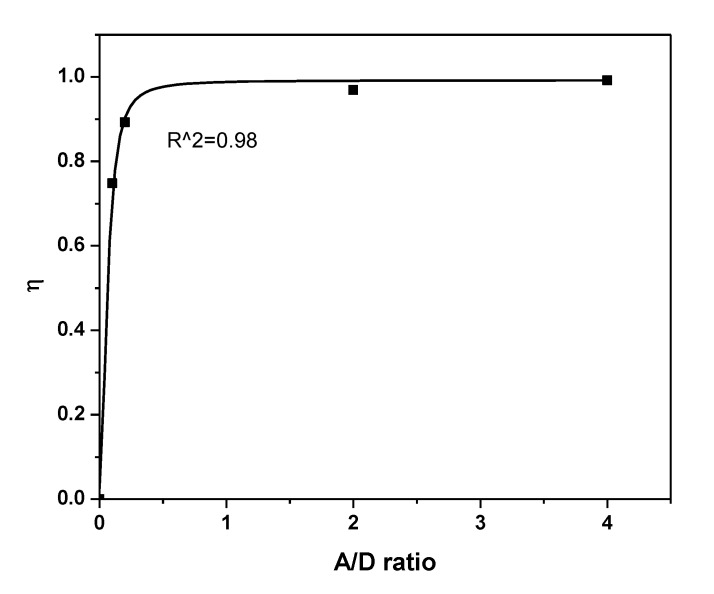
Energy transfer efficiency for 0-D Cs_4_PbBr_6_/MEH-PPV hybrid thin films.

**Figure 9:**
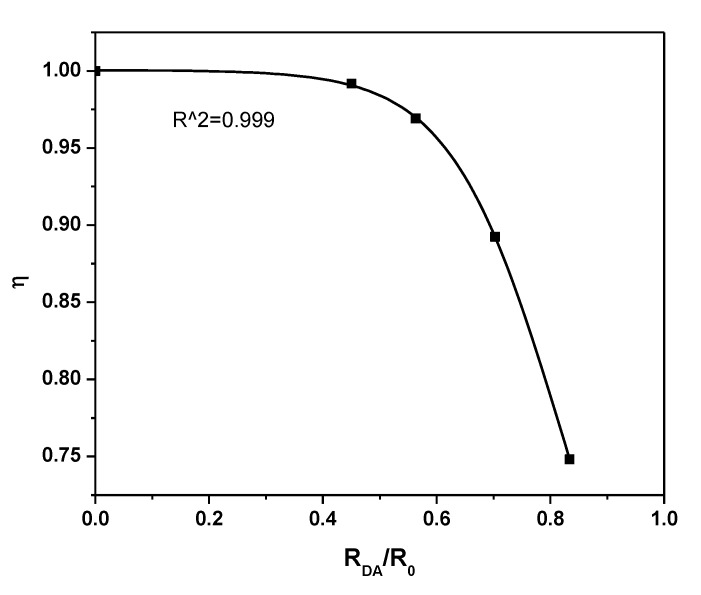
Energy transfer efficiency vis *R_DA_/R_0_* for 0-D Cs_4_PbBr_6_/MEH-PPV hybrid thin films.

The influence of the acceptor content on *P_DA_* and *η* is shown in Figure 7 and Figure 8, respectively. A gradual increase in both *P_DA_* and *η* with increasing acceptor content can be ascribed to the systematic decrease in the emission intensity (*I_DA_*). A systematic increase in *η* was observed until the A/D ratio reached 0.2, which then remained fixed with a maximum value at 0.99. An inflection point in *η* was detected at *R_DA_* = *R_o_*, as shown in Figure 9. At *R_DA_* < 0.5 *R_o_*, the value of *η* was close to unity and dramatically reduced for *R_DA_* > *R_o_*. Therefore, non-radiative energy transfer (Förster type) from 0-D Cs_4_PbBr_6_ to MEH-PPV happened with a higher probability for 10 Å < *R_o_* < 100 Å. Furthermore, the distance between molecules of the 0-D Cs_4_PbBr_6_ and MEH-PPV was less than 1.5 *R_o_*. These findings are in agreement with previous reports [17,31,32,33]. Since the distance between donor and acceptor is more than 1 nm, as presented in Table 2, the charge transfer in the current system can be neglected [34]. Moreover, despite the possibility of charge transfer according to the energy levels of both Cs_4_PbBr_6_ (HOMO = −5.73 eV, LUMO = −3.40 eV [35]) and MEH-PPV (HOMO = −5.3 eV, LUMO = −3.0 eV [36]), this mechanism can be neglected because of the strong overlap between donor emission and acceptor absorbance in addition to the enhancement of acceptor emission and decrease in that of donor [32,37]. In the Förster energy transfer process, once the Cs_4_PbBr_6_ irradiates with a light energy (hν), its oscillating dipole produces and resonates with the oscillating dipole of MEH-PPV. Consequently, the excited state energy can be transferred through space (dipole-dipole interaction) from the Cs_4_PbBr_6_ to MEH-PPV, without the exchange of electrons. The non-radiative energy transfer occurs once the Cs_4_PbBr_6_ returns to the ground state and then the MEH-PPV is brought to the excited state.

**Figure 10:**
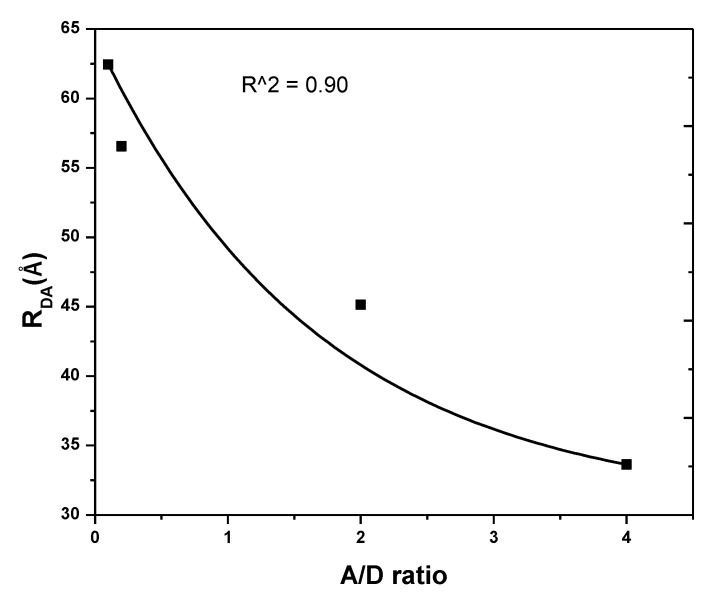
Energy transfer distance vis acceptor (A)/ donor (D) ratio for 0-D Cs_4_PbBr_6_/MEH-PPV hybrid thin films.

The energy transfer distance between D and A molecules (*R_DA_*) was based on *R_0_*, *I_D_*, and *I_DA_*. Figure 10 displays that as the A/D ratio increased from 0.1 to 4, the R_DA_ decreased from 62.45 to 33.64 Å (Table 2).

The *k_ET_* between a single D/A pair separated by *R_DA_* can be expressed in terms of *R_0_* as follows [38]:

(4) $$k_{ET}\;=\;\frac{1}{\tau_{D}}{\left(\frac{R_{o}}{R_{DA}}\right)}^{6}$$

As listed in Table 2, the values of *k_ET_* were significantly enhanced with the increasing A/D ratio. Moreover, the T_DR_ values (*k_ET_* + *τ_D_*^−1^) of the donor upon the addition of acceptor content in the hybrid thin films with various concentration ratios can be summarized in Table 2. As evidenced in earlier reports [17,30,32] and clearly detected in the present system of D/A hybrids, efficient energy transfer in the hybrids can be confirmed by the increases in both *k_ET_* and *T_DR_* upon increasing the acceptor content.

To suppress intermolecular transfer in the donor, the acceptor concentration should be much lower than *A_o_*, which is the acceptor concentration at which 76% of the energy was transferred [29]. The *A_o_* value of the MEH-PPV (~0.96 mM) was estimated based on the average value of *R_0_*. On the other hand, the distance between dipoles arising from the ground state to excited singlet state transition can be defined as A_π_ and derived from *k_r_* and *k_nr_*. No significant variation was observed in the value of *k_r_* (~0.085 ns^−1^) with increasing A/D ratio, whereas the value of *k_nr_* increased dramatically with the increasing A/D ratio. Subsequently, *A_π_* decreased with the increasing A/D ratio as listed in Table 1.

**Figure 11:**
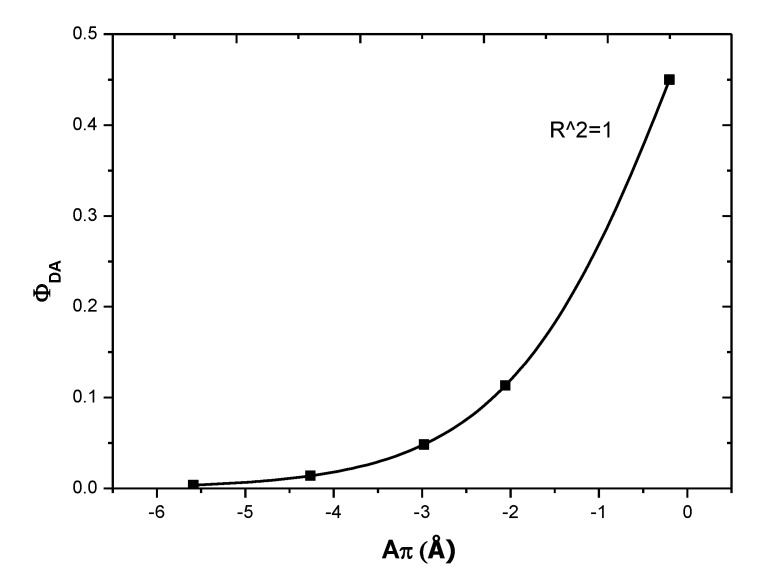
Relationship between *Φ_DA_* and *A_π_* in 0-D Cs_4_PbBr_6_/MEH-PPV hybrid thin films.

The exponential relationship between *Φ_DA_* and *A_π_*, as presented in Figure 11, shows that a new class of hybrids produced from organic/inorganic composites are highly fluorescent.

## 4. Conclusions

In the present work, hybrid thin films of 0-D Cs_4_PbBr_6_/MEH-PPV with various ratios were successfully prepared by a solution blending method. The form of the perovskite nanocrystals within a matrix of MEH-PPV in the hybrid thin films was determined from XRD and SEM analysis. No chemical interactions occurred between 0-D Cs_4_PbBr_6_ and MEH-PPV, as confirmed by XRD and absorption spectra analysis. The dominant energy transfer mechanism in the hybrids was Förster energy transfer, where the average critical distance of the energy transfer (*R_0_*) was 77.54 Å. The decrease in the emission of 0-D Cs_4_PbBr_6_ with the addition of MEH-PPV was due to both static and dynamic quenching, where 50% of the ground-state 0-D Cs_4_PbBr_6_ was complexed and thus, non-fluorescent at 20 µM of MEH-PPV. The hybrid of 0-D Cs_4_PbBr_6_/MEH-PPV is promising for use as an emissive layer in optoelectronic devices.

## Figures and Tables

**Table 1:** Optical Properties of the Donor/Acceptor Thin Films.

A/D Ratio	$\emptyset_{f}$	$k_{nr}\;{\left(\mathrm{ns}\right)}^{-1}$	$A_{\pi }\;(Å)$	$\tau_{f}\;(\mathrm{ns})$
0.0	0.450	0.104	−0.201	5.30
0.1	0.113	0.667	−2.057	1.33
0.2	0.0484	1.670	−2.979	0.57
2.0	0.0139	6.013	−4.261	0.164
4.0	0.0037	22.64	−5.586	0.044

**Table 2:** Energy Transfer Parameters of the Donor/Acceptor Thin Films.

A/D Ratio	J(λ) × 10^16^ (M^−1^ cm^−1^ nm^4^)	*R_0_* (Å)	*R_DA_* (Å)	$k_{ET}(\mathrm{ns}{-}^{1})$	*T_DR_* (ns^−1^)
0.1	4.97	74.87	62.45	0.56	0.75
0.2	7.66	80.47	56.55	1.57	1.76
2.0	7.49	80.17	45.15	5.91	6.10
4.0	4.88	74.65	33.64	22.55	22.74
